# TRIg: a robust alignment pipeline for non-regular T-cell receptor and immunoglobulin sequences

**DOI:** 10.1186/s12859-016-1304-2

**Published:** 2016-10-26

**Authors:** Sheng-Jou Hung, Yi-Lin Chen, Chia-Hung Chu, Chuan-Chun Lee, Wan-Li Chen, Ya-Lan Lin, Ming-Ching Lin, Chung-Liang Ho, Tsunglin Liu

**Affiliations:** 1 Department of Biotechnology and Bioindustry Sciences, National Cheng Kung University, Tainan City, Taiwan; 2 Molecular Diagnostic Laboratory, Department of Pathology, National Cheng Kung University Hospital, Tainan City, Taiwan; 3 Molecular Medicine Core Laboratory, Research Center of Clinical Medicine, National Cheng Kung University Hospital, Tainan City, Taiwan

**Keywords:** Sequence alignment, VDJ recombination, T-cell receptor, Immunoglobulin, RACE, Next-generation sequencing

## Abstract

**Background:**

T cells and B cells are essential in the adaptive immunity via expressing T cell receptors and immunoglogulins respectively for recognizing antigens. To recognize a wide variety of antigens, a highly diverse repertoire of receptors is generated via complex recombination of the receptor genes. Reasonably, frequencies of the recombination events have been shown to predict immune diseases and provide insights into the development of immunity. The field is further boosted by high-throughput sequencing and several computational tools have been released to analyze the recombined sequences. However, all current tools assume regular recombination of the receptor genes, which is not always valid in data prepared using a RACE approach. Compared to the traditional multiplex PCR approach, RACE is free of primer bias, therefore can provide accurate estimation of recombination frequencies. To handle the non-regular recombination events, a new computational program is needed.

**Results:**

We propose TRIg to handle non-regular T cell receptor and immunoglobulin sequences. Unlike all current programs, TRIg does alignments to the whole receptor gene instead of only to the coding regions. This brings new computational challenges, e.g., ambiguous alignments due to multiple hits to repetitive regions. To reduce ambiguity, TRIg applies a heuristic strategy and incorporates gene annotation to identify authentic alignments. On our own and public RACE datasets, TRIg correctly identified non-regularly recombined sequences, which could not be achieved by current programs. TRIg also works well for regularly recombined sequences.

**Conclusions:**

TRIg takes into account non-regular recombination of T cell receptor and immunoglobulin genes, therefore is suitable for analyzing RACE data. Such analysis will provide accurate estimation of recombination events, which will benefit various immune studies directly. In addition, TRIg is suitable for studying aberrant recombination in immune diseases. TRIg is freely available at https://github.com/TLlab/trig.

**Electronic supplementary material:**

The online version of this article (doi:10.1186/s12859-016-1304-2) contains supplementary material, which is available to authorized users.

## Background

T-cell receptor (TR) and immunoglobulin (Ig, also known as antibody) are essential in adaptive immune system as they recognize a wide variety of antigens, triggering immune response [1]. Each TR and Ig gene contains many coding regions, which are classified into variable (V), diverse (D, only in TRβ/δ and IgH genes) and joining (J) regions. For example, TRβ has 67 V, two D, and 13 J regions [2]. To recognize numerous antigens, TR and Ig genes undergo V(D)J recombination (i.e., selection and concatenation of a V, (D), and J region) at the DNA level for generating a large repertoire of structurally diverse receptors [3]. During recombination, the diversity is further enhanced via deletion and non-template addition of nucleotides within the so-called complementarity determining region 3 (CDR3), which is crucial for antigen recognition [4]. The knowledge of V(D)J recombination and CDR3 is thus important for studying immune response.

Several alignment tools have been available to analyze the complex recombination of TR and Ig genes, e.g., IMGT/V-QUEST [5]. After the introduction of next-generation sequencing (NGS), which generates a large amount of data, new tools for analyzing TR and Ig sequences are all geared toward faster speed. These include IMGT/HighV-QUEST [6], Decombinator [7], and the recent IgBLAST [8] and MiTCR [9]. Despite their distinct algorithms, all these tools do alignment only to the V(D)J regions instead of the whole gene to enhance speed. Software for subsequent analysis of diversity and clonality, e.g., tcR [10] and IMEX [11], are also available.

These tools have been quite useful for studying TR and Ig sequences, which are often prepared via a multiplex PCR approach [12, 13], in which multiple primers are designed to target different V and/or J regions. Such amplicon approaches are efficient in capturing regularly recombined TR and Ig genes, but likely suffer from amplification bias and miss non-regular TR and Ig sequences due to aberrant recombination in diseases [14, 15], cancerous cells [16, 17], or even healthy individuals [18]. Although amplification bias can be reduced [19], a complete removal of bias is still not warranted. To avoid amplification bias, 5′ RACE (rapid amplification of cDNA ends) strategy is promising [20] and has been applied in recent studies of immune repertoire [21, 22]. In addition, the strategy allows for detection of aberrant recombination and non-regular splicing events [23–25].

For RACE data, however, current tools can make mistake because they all assume regular recombination, which is not valid in many RACE sequences [26]. To fully utilize RACE data, we propose TRIg to handle non-regular TR and Ig sequences. Unlike all current programs, TRIg does alignment to the whole immune gene instead of only to the VDJ regions. With this strategy, TRIg avoids false V(D)J annotations to non-regular immune sequences. The strategy, however, is computationally challenging because full-length TR and Ig genes are relatively long and contain many repeats, which may result in multiple hits and the authentic hits need to be identified. The challenges have been properly managed in the TRIg pipeline.

On real RACE data, TRIg revealed several types of non-regular TRβ sequences, e.g., the expression of pseudo-gene J2-2P and concatenation of two J regions or J and intergenic regions. TRIg avoided false V(D)J annotation to those reads, thereby providing a more accurate description of immune repertoire. Accurate frequencies of V(D)J recombination have been used as biomarkers for health and disease [27, 28]. For those studies, an unbiased and accurate description of immune repertoire can be obtained using TRIg and RACE data. Besides, TRIg can unveil the rich behaviors of TR and Ig genes toward maturation, providing materials for a deeper understanding of the regulatory mechanisms. Therefore, we expect TRIg to benefit various immune researches.

## Implementation

### 5′ RACE 454 sequencing of human TRβ gene

Total RNA was extracted from the mononuclear cells in peripheral bloods of a healthy individual (male, age: 22). RNA was isolated using QIAGEN RNeasy mini kit (Qiagen, Germany). First strand cDNA was synthesized using SMARTer cDNA synthesis kit (Clontech) with a published TRBC GSC1 primer [29]. Then, SMART II oligonucleotide kit (Clontech Laboratories, US) was added to provide a 5′ template for RACE. The PCR protocol was as follows: 70 °C for 20 mins and 42 °C for 60 mins. The PCR mixture (25 uL) contained 1× PCR buffer for advantage 2 polymerase (Clontech), 0.125 mM of each dNTP, 10 pmole of UPM primer and TRBC GSC2 primer, 0.5 uL of polymerase mix, and 1 uL of undiluted first-strand cDNA. After first PCR, 1ul of the first-round product and an equimolar of three primers (Adaptor-UPM primer, Adaptor-TCRB-C1, Adaptor-TCRB-C2) were added for second PCR reaction (25 uL). After second PCR, the 520 bp products were visualized and purified by a DNA gel extraction kit (FavorPrep™). The purified products containing the primer were sequenced on Roche 454 GS Junior system following the manufacturer’s protocol.

### Sequencing data

The raw 454 reads were processed to remove primer segments and trimmed reads shorter than 100 bp were discarded. In addition to our own data, we obtained two public RACE data (Table 1) from NCBI Sequence Read Archive [30]. Again, processed reads wshorted than 100 bp were discarded.

**Table 1:** RACE data used in this study

Species; gene; sequencer	Read number	Mean length (bp)	SRA accession
Human; TRβ; 454	16,545	157	Our data
Human; TRβ; Illumina	1,522,640	209	SRR1544031
Mouse; IgH; 454	106,189	322	SRR934668-79; SRR934686-91

### TRIg pipeline

**Fig. 1:**
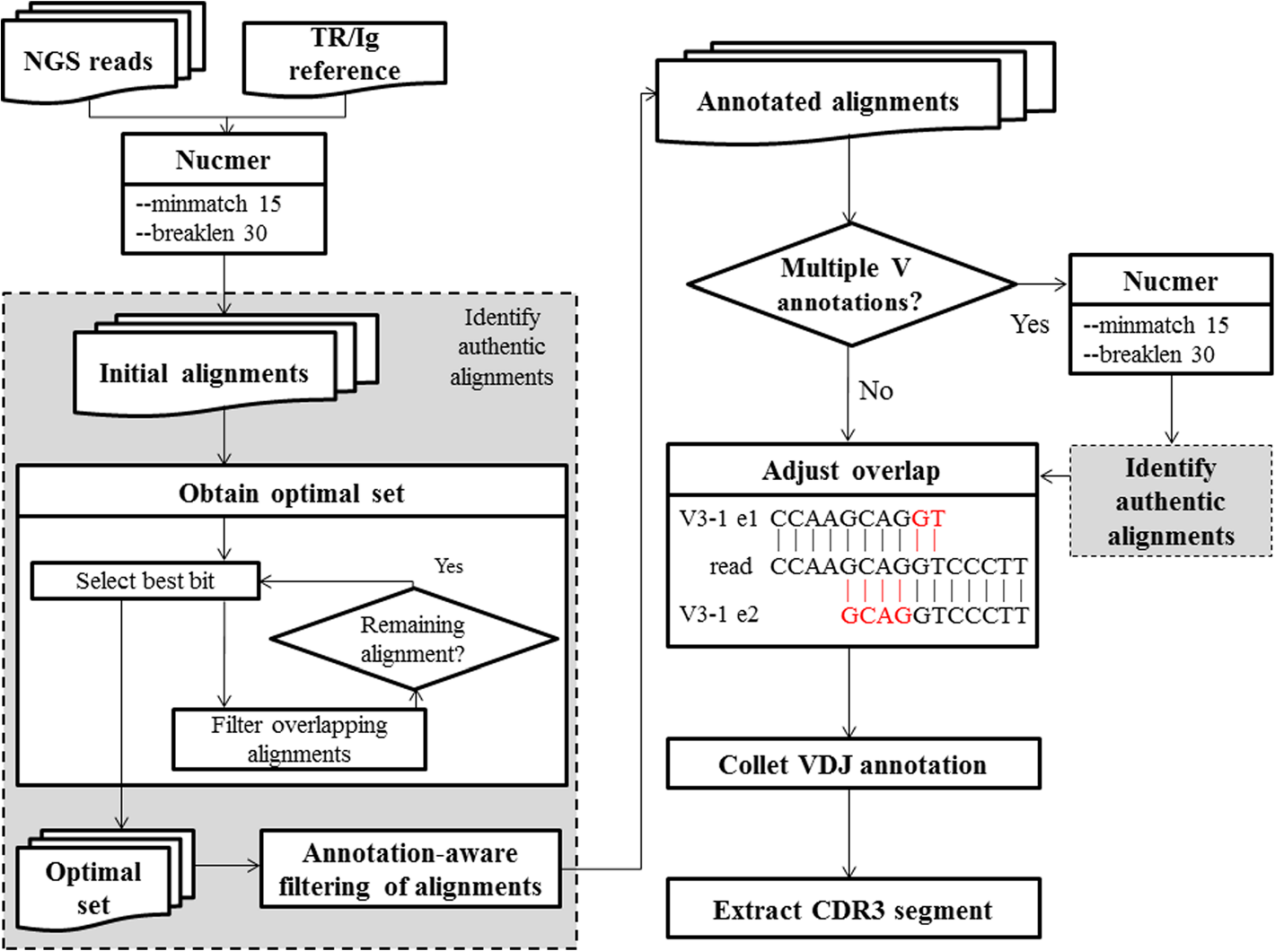
TRIg pipeline. TRIg starts by aligning TR or Ig reads to the corresponding reference using nucmer. Because many initial alignments overlap, thus not authentic, optimal sets of alignments are obtained using a heuristic iterative approach. Some of the optimal alignments are further filtered based on the VDJ annotations. If more than one V annotation survives the filtering, TRIg intends to extend the alignments by relaxing the breaklen parameter of nucmer, followed by re-identification of authentic alignments. Because the resulting alignments may still overlap by few bases, the overlapping bases are trimmed (in *red*). Finally, VDJ annotations are collected and the CDR3 segments are extracted. Please see main text for detailed descriptions

TRIg aligns reads to the TR/Ig gene in four steps (Fig. 1): (1) initial alignments to the whole TR/Ig reference using nucmer [31, 32], (2) obtaining optimal sets of alignments, (3) filtering alignments based on VDJ annotation, and (4) realigning the reads with multiple V annotations. The resulting alignments are further adjusted to eliminate overlapping bases between alignments. Adjusted alignments are then associated with VDJ regions and the corresponding CDR3 segments are extracted. These steps are explained in details as follows.

1. *Initial alignments of reads to the whole TR/Ig reference using nucmer*
  TRIg uses nucmer (v3.1) to do initial alignments to the whole TR/Ig reference. To allow multiple hits to repetitive regions in the reference, nucmer is set to use all anchor matches regardless of their uniqueness.
2. *Obtaining optimal sets of alignments*
  For each read, many initial alignments overlap a longer one by more than half of the aligned regions, suggesting their invalidity. TRIg applies an iterative approach to filter invalid overlapping alignments. First, the alignment with the maximal score (based on nucmer’s scoring scheme) is selected to be in the optimal set. Second, all alignments that overlap the best one are filtered. Third, from the remaining alignments select the next best alignment to be in the optimal set and do the filtering. This procedure is repeated until no alignment remains. Note that there can be more than one best alignment with equal score and all of them are included in the optimal set of alignments.
3. *Annotation-aware filtering of alignments*
  Equally good alignments can be further reduced using VDJ annotation. For example, a read segment can be aligned equally well to the first exon of V4-2 and V4-3 while another read segment is best aligned to the second exon of V4-3. This suggests that the alignment to the first exon of V4-2 should be filtered. In this step, TRIg also filters alignments of putative CDR3 segments. To identify a putative CDR3 alignment, TRIg searches for an alignment flanked by the second exon of a V region on one side and J exon on the other. An identified alignment survives if the segment is from a D or J region or the alignment is long (≥60 bp) and is otherwise filtered. In addition, short (<40 bp) and inferior (identity <95 %) alignments to intergenic regions are filtered in this step.
4. *Realigning reads with multiple V annotations*
  After the above filtering, more than one V annotation can still remain for some reads. To differentiate multiple V alignments, TRIg attempts to extend the alignments further outward by relaxing the nucmer parameter breaklen. In some cases, one of the extended alignments stands out better than others and is then selected.
5. *Eliminating overlapping bases*
  Alignments overlapping by only few bases can survive the above filtering processes. For those alignments, TRIg determines an optimal cut point, beyond which the overlapping parts are trimmed (Fig. 1). An optimal cut point is found when the trimmed alignments give a maximal total score. To favor alignments to exons, each aligned base within a VDJ region receives one more point. For overlapping alignments that span two exons of a V gene, this helps to stop alignment at the exon boundary.
6. *VDJ annotation and extraction of CDR3 sequences*
  For each read, the resulting alignments are associated with either a V, D, J, C (constant) or intergenic region. To extract CDR3 sequences, TRIg searches for pairs of alignments annotated as the last exon of a V gene and a J region respectively. Once found, the starting and ending positions of CDR3 region on the reads are determined according to the definition in IMGT website and the CDR3 segments are extracted.

### Related programs

Both Decombinator (v2) and IgBLAST (v1.4.0) were downloaded and run with default parameters. IMGT/HighV-Queset (v1.3.1) analyses were performed on the IMGT website using the default settings.

## Results

TRIg was compared to Decombinator, IgBLAST, and IMGT/HighV-Quest (abbreviated as IMGT hereafter) using our own RACE data of a healthy individual and two public data (Table 1). A healthy individual was selected to alleviate complication by disease. Public data were used to show generality of results in different implementations of RACE experiments and sequencing approaches. Only annotations of V and J regions were compared because D regions are relatively short and nucleotide modifications often occur at the exon boundaries. In addition to VJ annotations, annotations of non-VJ segments (i.e., constant C or intergenic segments) were considered. Note that only TRIg could give a non-VJ annotation and there could be more than one V or J annotation by all programs except Decombinator. The results for each data were described as follows.

### Our RACE data of human TRβ gene

Among the four programs, IgBLAST was the most sensitive as it annotated 99.6 % of the reads (Table 2). If non-VJ annotations were included, TRIg became the most sensitive. In contrast, Decombinator and IMGT made annotations to 29.1 and 34.5 % of reads, respectively.

**Table 2:** Number of VJ annotations by four programs

Data	Decombinator	IgBLAST	IMGT	TRIg (including non-VJ annotations)
Our data	4807	16,487	5711	12,260 (16,538)
SRR1544031	1,190,792	1,521,612	1,232,628	1,456,541 (1,517,758)
SRR9346 (68-79;86-91)	N.A.	105,850	64,819	87,286 (106,111)

Among the reads annotated by TRIg but not Decombinator, most (92.7 %) did not contain both a V and a J segment. This is reasonable because Decombinator requires the presence of both a V and a J segment, therefore did not make annotation to reads without a regular V-J structure. TRIg considered the remaining 862 reads as regular, but Decombinator still did not make annotation. This may be explained by the fact that Decombinator looks for specific V and J segments instead of matches to any part of V and J sequences. Among the reads annotated by TRIg but not IMGT, most (98.3 %) did not contain a V segment. Again, this is reasonable because IMGT requires the presence of a V segment. These results indicate that Decombinator and IMGT were less sensitive than TRIg mainly because they did not annotate non-regular TRβ sequences.

To examine the consistency of annotations by TRIg and a program, annotations were split into four categories: (1) identical --- when two annotations were the same, (2) extra --- when the program made an additional V or J annotation than TRIg, (3) missing --- when the program missed a V or J annotation by TRIg, and (4) distinct --- when the program and TRIg made distinct V or J annotations. In addition to the four categories, non-VJ annotations by TRIg were included in statistics.

When both TRIg and Decombinator made annotations, they agreed in most (91.4 %) cases (Table 3). For the remaining reads, Decombinator either missed one of the multiple V annotations by TRIg (5.3 %) or gave a distinct annotation (3.2 %). The multiple V annotations by TRIg should be valid because the corresponding alignments were equally good. Decombinator could not reveal those possibilities because it always reports only one V and one J annotation.

**Table 3:** Consistency of VJ annotations to our data

TRIg v.s.	Identical	Extra	Missing	Distinct	Non-VJ
Decombinator	4394	1	257	155	0
IgBLAST	5733	2028	30	4411	4278
IMGT	5466	131	68	45	1

**Fig. 2:**
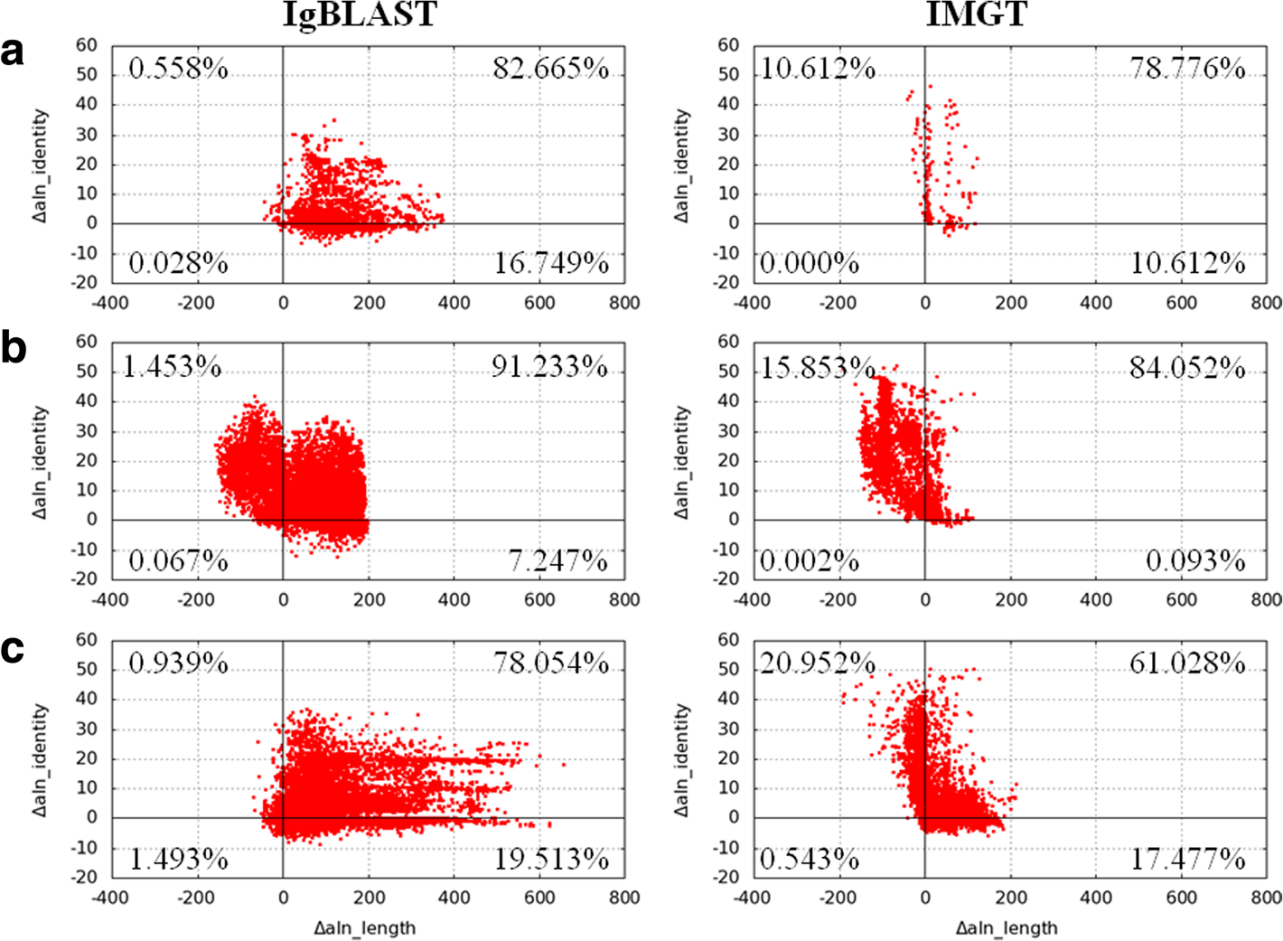
Comparison of immune sequence alignments by different tools. Differences in length (x-axis) and identity (y-axis) of non-identical alignments by IgBLAST and TRIg (*left column*) and IMGT and TRIg (*right column*) for (**a**) our 454 data of human TRβ gene, (**b**) public Illumina data of human TRβ gene, and (**c**) public 454 data of mouse IgH gene. The differences were obtained by subtracting IgBLAST’s or IMGT’s values from TRIg’s values. Thus, dots in the first quadrant clearly indicate better alignments by TRIg. The validities of alignments in the second and fourth quadrants are less clear. However, for most dots in the two quadrants, TRIg’s annotations are more convincing because TRIg’s alignments are much longer or the identities much higher. Note that the dots may fall on top of each other, this explains the seemingly fewer dots than indicated in the first quadrant of (**b**)

IMGT was also quite consistent with TRIg because 95.7 % of their annotations were identical to TRIg’s annotations (Table 3). When TRIg and IMGT disagreed, TRIg’s annotations were usually more convincing because TRIg gave better alignments than IMGT did for 78.8 % of the non-identical annotations (Fig. 2a). Detailed examinations revealed that IMGT missed some J annotations because it did not allow gaps in J alignments. In addition, IMGT sometimes reported alignments of relatively low identities (50–80 %). Such IMGT alignments could be slightly longer than the TRIg’s alignments; however, the low identities did not support the IMGT’s annotations (Fig. 2a). These results suggest that TRIg is more robust than IMGT even for regular TRβ sequences. For non-regular TRβ sequences, e.g., those with only a J segment according to TRIg, IMGT might mistake the intergenic segment in the upstream of a J region for a V region. Similarly, a CDR3 segment in the downstream of a V region could be mistaken as a J region by IMGT, which explained some of the IMGT’s extra annotations.

Compared to Decombinator and IMGT, IgBLAST was more different from TRIg as only 34.8 % of the annotations by IgBLAST and TRIg were identical (Table 3). For the extra annotations by IgBLAST, most (98.2 %) reads did not contain a V segment according to TRIg. This could be attributed to IgBLAST’s low stringency in V alignments, which resulted in false V annotations to non-V segments. For example, the upstream or part of J segments of non-regular reads could be mistaken for V segments by IgBLAST. Similarly, for most (98.3 %) of the distinct annotations, IgBLAST reported a V annotation while TRIg considered the read without a V segment. For all non-identical annotations, TRIg gave better alignments than IgBLAST for 82.7 % of reads (Fig. 2a).

In addition to our data, a public RACE data of human TRβ gene from a 454 sequencer (NCBI SRA accession SRR941034) was analyzed. Many statements for our data still held for that data (Additional file 1: Supplementary Results). For example, TRIg gave a better alignment than IMGT and IgBLAST did for a majority of the non-identical annotations. The similar pattern of results suggests that the presence of non-regular TRβ sequences is common in RACE approach and the performance of all programs on 454 data is consistent.

### Public RACE data of human TRβ gene

To examine the usefulness of TRIg on data from a different sequencing platform, a public RACE data of human TRβ gene generated on an Illumina sequencer was analyzed. Compared to our data, a major difference in the results was that IgBLAST and TRIg were much more consistent in annotating this Illumina data (Table 4), which is reasonable as TRIg considered a higher percentage of reads (80.4 v.s. 34.8 %) as regular. For the ~20 % non-regular reads, TRIg considered most (95.3 %) without a V segment while IgBLAST reported V alignments. For reads with non-identical annotations, TRIg’s alignments were clearly better than IgBLAST’s and IMGT’s alignments in 91.2 and 84.1 % of cases, respectively (Fig. 2b). For some reads in this data, IgBLAST and IMGT reported relatively longer but of much lower identity alignments compared to TRIg. The low alignment identities by IgBLAST and IMGT were likely the results of low sequencing quality (Additional file 1: Figure S3). Facing low quality reads, IgBLAST and IMGT might still output low identity alignments while TRIg did not.

**Table 4:** Consistency of VJ annotations to the SRR1544031 data

TRIg v.s.	Identical	Extra	Missing	Distinct	Non-VJ
Decombinator	1,111,024	66	42,448	37,254	0
IgBLAST	1,226,408	42,952	343	186,698	60,850
IMGT	1,191,269	38,376	796	1204	645

### Non-regular VDJ recombination

In the two RACE data of human TRβ gene, several classes of non-regular VDJ recombination were observed. According to TRIg, 65.7 % of the reads in our data were non-regular (i.e., did not contain both a V and a J segment). Among those, the most abundant class were sequences containing a J but not a V segment. For those reads, the J segment either extended to upstream of the J region (77.3 %) or was concatenated to a D segment (14.0 %), a constant C segment (5.3 %), an intergenic segment (2.7 %), or another J segment (0.8 %). Note that the extension to upstream of a J region could be long enough to cover a neighboring J segment, among which most were a stretch from J2-2P to J2-3. When a J segment was concatenated to a D segment, most of the D segments were longer (≥30 bp) than the D regions (≤16 bp) and extended only into upstream of the D regions. This suggests suppressed recombination of V and DJ segments. The second most abundant class of non-regular reads (34.8 %) in our data was segments from only intergenic regions and most (98.4 %) of the intergenic segments were relatively long (≥100 bp). Interestingly, most (97.3 %) of the intergenic segments appeared within a region upstream of TRBD1, suggesting aberrant recombination between D and J segments. In addition to the two classes, some reads contained only C segment while others contained only a V segment. Most of the above non-regular recombination was also observed in another 454 data (Additional file 1: Supplementary Results). Besides, recombination between a TRβ segment and non-TRβ gene was detected in that data.

In the Illumina data, the percentage of non-regular reads (19.6 %) was much lower but similar observations of non-regular recombination were made. Besides, a majority of reads, including regular reads, contained a C segment, which appeared likely because the primer targeted more downstream of the C region.

### Public RACE data of mouse IgH gene

For the mouse IgH data, TRIg was compared only to IgBLAST and IMGT because Decombinator had not yet supported alignments to mouse IgH gene. For this dataset, 44.0 and 68.9 % of annotations by IgBLAST and IMGT were identical to TRIg’s annotations, respectively (Table 5). Note that both IgBLAST and IMGT included provisional versions of V genes, e.g., IgHV1S11 and IgHV1S137, which did not appear in the TRIg’s reference set. Because those annotations could not be compared fairly, the reads annotated as a provisional V gene by IgBLAST or IMGT and as a V gene by TRIg were excluded from the following analyses. This excluded about 8.9 % of the total reads when TRIg was compared to IgBLAST and IMGT. Among the remaining non-identical annotations, TRIg achieved a clearly better alignment than IgBLAST and IMGT for 78.1 and 61.0 % of the reads, respectively (Fig. 2c).

**Table 5:** Consistency of VJ annotations to the SRR9346(68-79;86-91) data

TRIg v.s.	Identical	Extra	Missing	Distinct	Non-VJ
IgBLAST	46,496	6601	5799	28,144	18,740
IMGT	44,684	2736	6328	11,057	14

Note that IgBLAST or IMGT gave an annotation not present in the reference set of TRIg to 8.9 % of the reads, resulting in non-identical annotations

For the rest non-identical annotations between TRIg and IgBLAST, TRIg gave a much longer (by ≥40 bp) but of slightly lower identity (by <2 %) alignment in a majority (71.4 %) of cases, suggesting validity of the TRIg’s annotations in general. However, there were also cases where TRIg’s alignment was only slightly longer. This can be attributed to the different reference sets used by TRIg and IgBLAST. IgBLAST includes multiple versions of V, D, or J genes, which differ by few nucleotides, in the reference set. In contrast, TRIg uses only one single reference sequence. Therefore, IgBLAST’s alignments could be slightly better than TRIg. The different reference sets used by IgBLAST and IMGT could also explain why IgBLAST sometimes gave a longer and of higher identity alignment than TRIg did. Most of these statements also hold when TRIg was compared to IMGT. However, we found that IMGT sometimes gave alignments with a relatively low identity compared to IgBLAST and TRIg (Fig. 2c).

For this dataset, TRIg considered 38.2 % of the reads as non-regular. Among those, 49.7 % were reads containing only J and constant segments. Interestingly, most reads contained more than one constant segment from the same region, suggesting artifacts of primer concatenations.

## Discussion

### Applicability of the computational programs

TRIg is a stand-alone program written in Perl and is designed for Linux system. Both Decombinator and IgBLAST also offer stand-alone programs for analyzing TR sequences, but Decombinator does not work for Ig sequences. In contrast, IMGT can only be run through the webserver. Among those programs, Decombinator was most efficient in terms of run time (Table 6); however, it was also the least sensitive in terms of VJ annotation. TRIg ran faster than IgBLAST and IMGT and can be easily accelerated with multiple processors. For non-regular TR and Ig sequences in the RACE data, IgBLAST was over-sensitive and gave false VJ annotations. In contrast, IMGT was more conservative and made less false VJ annotations; however, it did not examine the details of non-regular sequences. Because non-regular TR and Ig sequences are not uncommon in RACE data, TRIg is the program of choice for analyzing RACE data. Although RACE approach is less efficient than the common multiplex PCR approach in probing regular V(D)J recombination, it avoids primer bias and offers a more accurate estimation of immune repertoire. As the cost of NGS keeps decreasing, the lower efficiency of RACE approach will be less a concern. More importantly, accurate description of immune repertoire should be the top priority.

**Table 6:** Run time of four programs on the three data

Run time	Decombinator	IgBLAST	IMGT	TRIg
Our data	0 m 8 s	4 m 43 s	84 m	0 m 15 s
SRR1544031	11 m 41 s	653 m 32 s	N.A.^a^	42 m 10 s
SRR9346(68-79;86-91)	N.A.	135 m 01 s	155 m	25 m 17 s

IMGT jobs were done on the webserver. The rest tools were run using only one processor (800 MHz). ^a^Not available because the data was split into 11 files and the total run time did not reflect the true run time

### Nucmer is suitable for initial alignments of TR and Ig sequences

The initial alignments of TRIg can be performed using various tools, e.g., BLAST, BLAT, Bowtie2, etc. BLAT seems suitable for aligning TR and Ig sequences because it is designed for aligning cDNA to the genome and V(D)J recombination can be thought of as a special kind of splicing events. However, we found that BLAT sometimes aligned a V segment incorrectly when similar V exons exist (data now shown). The presence of similar exons is not common for most genes, but is an issue for TR and Ig genes. Therefore, the general purposed BLAT is less suitable for TR and Ig sequences. Bowtie2 is designed to treat NGS data efficiently. With the default options, Bowtie2 ran as fast as nucmer on our data but was slower than nucmer on the public data. The default options, however, only report the best hit of end-to-end alignments, which do not match the nature of TR and Ig sequences. To align segments from different loci of the gene, one must enable the “local” option of Bowtie2 and ask it to report multiple hits. These options doubled or even tripled the run time, making Bowtie2 less efficient than nucmer on TR and Ig sequences. Similarly, BLAST spent more time to finish initial alignments compared to nucmer.

### Applications of TRIg

TRIg allows for quantifying amplification bias of a multiplex PCR approach when a RACE approach is also applied. Note that multiplex PCR can be applied at the gDNA or mRNA (i.e., cDNA) level [20]. A gDNA data reveals the counts of distinct T or B cells. In contrast, a cDNA data measures the expressions of T or B cell receptor genes in all distinct T or B cells. From a functional point of view, cDNA provides better insight as mRNAs are closer to functional proteins. Since RACE is also applied at the cDNA level, comparisons should be made to a multiplex PCR approach at the cDNA level.

Another application of TRIg is to dissect non-regular recombination in immune diseases. For example, in T cell leukemia cells two J segments could recombine in a head-to-head manner [16, 17]. Such aberrant recombination can be recognized readily by TRIg. In T cell acute lymphoblastic leukemia, chromosomal translocation could result in fusion of a TR gene to a non-TR locus [33, 34]. For such non-regular TR sequences, TRIg will not annotate the non-TR segment and the non-TR locus can be further determined via alignment against the whole genome.

Besides, TRIg allows for studying the role of non-regular recombination in immune system. In the RACE data, many non-regular TR and Ig sequences suggest incomplete VDJ recombination. For example, a J segment was recombined to a D segment, but not further to a V segment. One explanation of such incomplete recombination is the phenomenon of allelic exclusion [35]. For a diploid genome, allelic exclusion interrupts the recombination of the second allele of a TR gene when the first allele is recombined successfully. This results in only a single type of TR on the surface of a T cell. It is interesting that the incompletely recombined TR alleles are also transcribed and their roles in immune system can be investigated using TRIg.

## Conclusions

TRIg is the first alignment pipeline for analyzing TR and Ig sequences while taking into account non-regular V(D)J recombination. This unique feature is particularly useful for analyzing RACE data, in which many TR or Ig sequences are not regular. Applying TRIg on RACE data will give accurate description of immune repertoire. Therefore, TRIg should benefit researches of immune system and improve the prediction of various immune diseases.

## Additional file

Additional file 1: Is a pdf file containing Supplementary Results, Tables S1-S3 and Figures S1-S2 as described below. **Table S1** PCR primer for 5′ RACE and the primer and barcode (MID) sequences used in 454 sequencing. **Table S2** Number of VJ annotations by four programs to the SRR941034 data. **Table S3** Consistency of VJ annotations to the SRR941034 data. **Figure S1** Flow of 5′ RACE experiment. **Figure S2** Comparison of alignments by different programs for the SRR941034 data. Please check Fig. 2 of the main text for explanations. **Figure S3** Base quality of reads in the (a) first and (b) second quadrant of Fig. 2b of the main text when TRIg is compared to IgBLAST and IMGT. (PDF 312 kb)

## Abbreviations

CDR: Complementarity determining region
Ig: Immunoglobulin
NGS: Next-generation sequencing
RACE: Rapid amplification of cDNA ends
TR: T-cell receptor
