# Supplementary material for: TRIg: a robust alignment pipeline for non-regular T-cell receptor and immunoglobulin sequences
Source: BMC Bioinformatics. 2016 Oct 26;17:433. doi: 10.1186/s12859-016-1304-2 (PMC5080739; doi:10.1186/s12859-016-1304-2)
Supplement: Additional file 1: — Is a pdf file containing Supplementary Results, Tables S1-S3 and Figures S1-S2 as described below. Table S1 PCR primer for 5′ RACE and the primer and barcode (MID) sequences used in 454 sequencing. Table S2 Number of VJ annotations by four programs to the SRR941034 data. Table S3 Consistency of VJ annotations to the SRR941034 data. Figure S1 Flow of 5′ RACE experiment. Figure S2 Comparison of alignments by different programs for the SRR941034 data. Please check Fig. 2 of the main text for explanations. Figure S3 Base quality of reads in the (a) first and (b) second quadrant of Fig. 2b of the main text when TRIg is compared to IgBLAST and IMGT. (PDF 312 kb) [file 12859_2016_1304_MOESM1_ESM.pdf]

Supplementary data

Sheng-Jou Hung, Yi-Lin Chen, Chia-Hung Chu, Chuan-Chun Lee, Wan-Li Chen, Ya-Lan Lin, Min-Ching Lin, Chung-Liang Ho, Tsunglin Liu*

Supplementary Results

Public RACE data of human TRβ gene

To address the concern whether the presence of non-regular TRβ sequences is unique to our approach, we analyzed a public RACE data of human TRβ gene generated by a 454 sequencer (NCBI SRA accession SRR941034). For this dataset, IgBLAST was the most sensitive as it did not annotate only 0.5% of the reads (Table S2). In contrast, TRIg did not annotate 8.5% of the reads. For those reads, the V alignments by IgBLAST were either short (<20 bp in 71.0% of cases) or of low identities (<0.8 in 97.1% of alignments ≥30 bp). This again suggests that IgBLAST is over-sensitive for non-regular VDJ sequences. Compared to our data, the overall consistency of annotations (Table S3) showed a similar pattern except that IgBLAST was more consistent with TRIg (71.2% of the annotations were identical) and there were relatively more non-identical annotations in the non-VJ categories. The better consistency between IgBLAST and TRIg was reasonable because the percentage of reads without a V segment in this dataset (28.0%) was smaller than that in our data (64.5%) according to TRIg. Many statements for our data still held for this dataset. For example, TRIg gave a better alignment than IMGT did for a majority of the non-identical annotations (Figure S2). The similar pattern of results suggests generality of these tools on RACE data of human TRβ gene.

However, there were still distinctions in the results. For example, IgBLAST gave a longer but of lower identity alignment for 38.5% of the reads with non-identical annotations in this dataset (Figure S2), much higher than the <1% in our data. For most (97.7%) of those reads, TRIg identified only a segment in the constant C region while IgBLAST reported V and/or J alignments. The V and J alignments by IgBLAST were either short (<20 bp in 79.4% of the cases) or of a low identity (<0.8 in 97.1% of alignments ≥30 bp); therefore were less convincing. Similarly, IgBLAST reported an extra J alignment in 88.3% of the extra annotations and 96.8% of the J alignments were short (<20 bp). These again suggest the over-sensitivity of IgBLAST. In contrast, most of the constant C

segments identified by TRIg were from the same C locus, and they were likely primer sequences used in the RACE approach. Thus, TRIg's annotations for those reads were more convincing.

Another distinction was that between IgBLAST and TRIg, relatively more non-identical annotations appeared in the non-VJ category. For 15704 reads in the non-VJ category, TRIg found only a short C segment, which again was likely primer sequence. This suggests that the remaining segments were from non-TRB genes. To confirm the statement, the 15704 reads were aligned to human genome (h38) using BLAT (v35) and the best alignments were selected. The best alignments of 15528 reads fell outside TRβ gene locus. Along this line, we aligned all the reads to the human genome and found that 20.2% contained a segment that could be aligned to a non-TRβ locus. In contrast, only 0.2% of reads in our data could be aligned to a non-TRβ locus. This explained why TRIg failed to annotation 8.5% of reads and some reads were not fully aligned.

In the public RACE data of human TRβ gene, 42.7% of reads were non-regular, among which the most abundant class (52.8%) were sequences containing only a V segment. For those reads, it is possible that the sequencing started from the V segments but was not long enough to reach the J segments. The second most abundant class (26.9%) of non-regular reads were sequences containing only a short C segment. This echoed our discovery that a good portion of this public data contained sequences from a non-TRβ locus and a C segment (putative RT-PCR artifact) was concatenated to the non-TRβ segment. The next two abundant classes were non-regular reads with a C segment connecting only to a J segment (9.0%) or an intergenic segment (6.2%), respectively. Interestingly, most of the intergenic segments were from two TRβ loci, one in the upstream of TRBD1 and the other in the upstream of TRBC1, suggesting non-regular splicing events.

Supplementary Tables

**RT-PCR**	
5' RACE primer	AAGCAGTGGTATCAACGCAGAGTACATGGG
TCRB-GSC1	CACGTGGTCGGGGWAGAAGC

**First PCR**			
10x UPM	Long (0.4uM)<br>CTAATACGACTCACTATAGGGCAAGCAGTGGTATCAACGCAGAGT<br>Short (2uM)<br>CTAATACGACTCACTATAGGGC		
TCRB-GSC2	GGGTGGGAACACCTTGTTCAGGT		
**Nested PCR**			
UPM primer	CTAATACGACTCACTATAGGGC		
Adaptor-UPM primer (Seq primerA-key-MID1-UPM)<br>CGTATCGCCTCCCTCGCGCCATCAGACGCTCGACACTAATACGACTCACTATAGGGC			
TCRB-C1	GGGTGGGAACACCTTGTTCAGGT		
Adaptor-TCRB-C1 (Seq primerB-key-MID1-TCRB-C1)<br>CTATGCGCCTTGCCAGCCCGCTCAG ACGAGTGCGTGGGTGGGAACACCTTGTTCAGGT			
TCRB-C2	GGGTGGGAACACCTTTTTCAGGT		
Adaptor-TCRB-C2 (Seq primerB-key-MID1-TCRB-C2)<br>CTATGCGCCTTGCCAGCCCGCTCAG ACGAGTGCGTGGGTGGGAACACCTTTTTCAGGT			
**454 Junior MID**			
MID1	ACGAGTGCGT	MID8	CTCGCGTGTC
MID2	ACGCTCGACA	MID9	TAGTATCAGC
MID3	AGACGCACTC	MID10	TCTCTATGCG
MID4	AGCACTGTAG	MID11	TGATACGTCT
MID5	ATCAGACACG	MID12	TACTGAGCTA
MID6	ATATCGCGAG	MID13	CATAGTAGTG
MID7	CGTGTCTCTA	MID14	CGAGAGATAC

Table S1. PCR primer for 5' RACE and the primer and barcode (MID) sequences used in 454 sequencing.

Data	Decombinator	IgBLAST	IMGT	TRIg (including non-VJ annotations)
SRR941034	55,551	140,485	98,544	108,569 (129,198)

Table S2. Number of VJ annotations by four programs to the SRR941034 data.

TRIg v.s.	Identical	Extra	Missing	Distinct	Non-VJ
Decombinator	53,000	40	659	1852	0

IgBLAST	91,634	9,558	436	6,831	20,263
IMGT	87,403	9,234	1,278	531	87

Table S3. Consistency of VJ annotations to the SRR941034 data.

Supplementary Figures

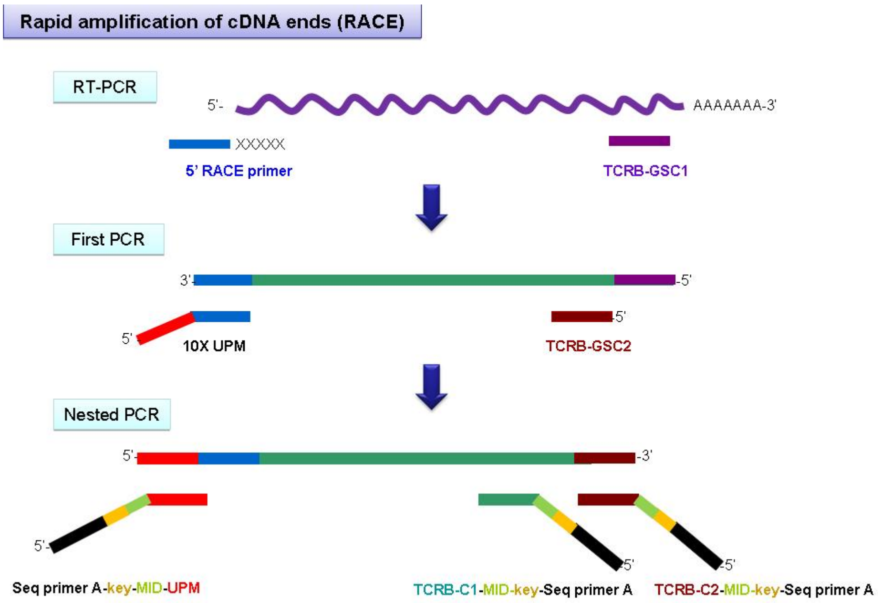

Figure S1. Flow of 5' RACE experiment. Please see Table S1 for the primer and MID sequences.

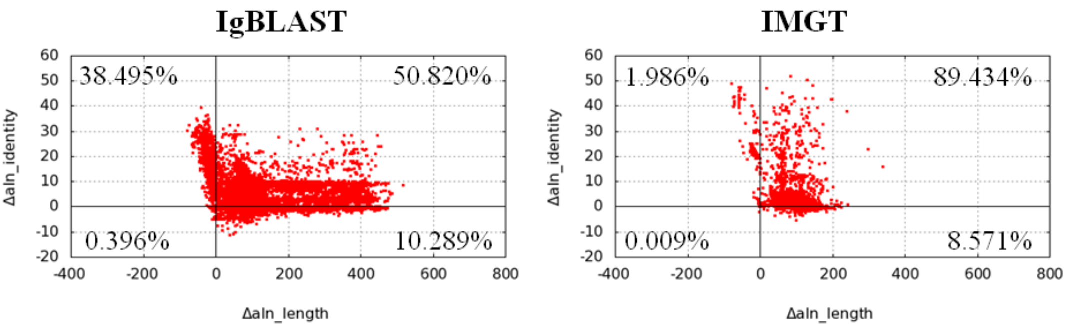

Figure S2. Comparison of alignments by different programs for the SRR941034 data. Please check Figure 2 of the main text for explanations.

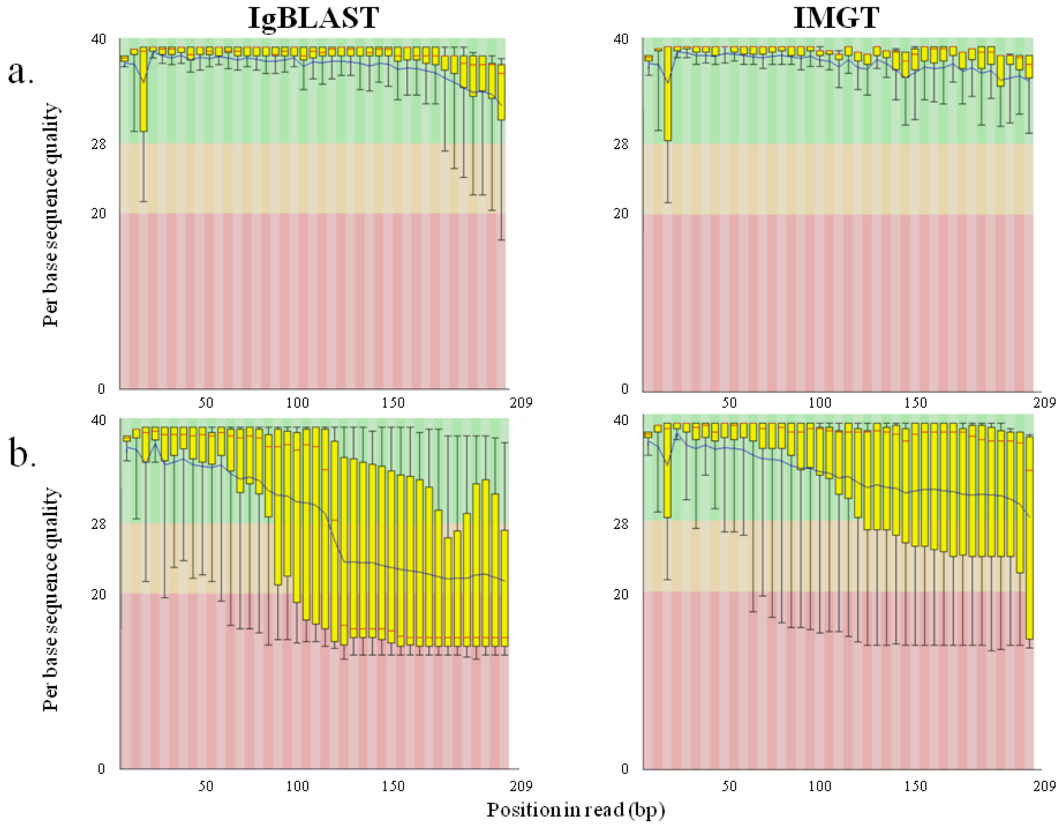

Figure S3. Base quality of reads in the (a) first and (b) second quadrant of Figure 2b of the main text when TRIg is compared to IgBLAST and IMGT.
